# Don’t Try to Do Too Many Things

**Published:** 1875-06

**Authors:** 


Don't Try to do too Many Things.—In doing a thing there are several points to be considered, as the resistance to be overborne, the strength we have, and whether, if we are able to do it, the necessary exertion is not better reserved for something else. A man may be able to get a barrel of flour up stairs, or pull on a pair of boots considerably too small, and keep a bottle of liniment by him for a week after in consequence. A woman with an arm full of bundles or a heavy baby, may succeed in catching a horse-car ten rods ahead; and pay for her triumph by vertigo, or something worse. She may take up, whip, and put down a half dozen carpets, in addition to the Spring scrubbing and general housework, as some ambitious workers do; but such misspent force usually results in the speedy introduction of a step-mother into the family. Years ago we knew a family that for three generations had drawn all the water for household use by a well-sweep nine and one-half rods distant, when there was nothing to prevent the digging of a well at the house and the drawing of the water by a pump. About the same time we also had knowledge of a skillful wall-layer, whose labor was in constant demand, who worked by the week at cutting up old stumps for his winter fuel when he could have bought three times the amount of good cut fire-wood with the money readily acquired in his appropriate calling. Our mind also goes back to the period when there lived a man of rare mechanical genius, who could have achieved marked success by steady aims and well-directed industry, but who yet so misapplied his acknowledged capacity as to prove a signal failure in life, becoming bankrupt in fortune and even in name. Indeed, it is easy to see that many people are wasting much time and frequently doing much damage, by misdirecting their physical powers.

---

All amusements and recreations are lawful and innocent, which tend to promote health of body, vigor of mind, purity of soul, and thus qualify for a better discharge of higher and more important duties.

---

In getting rich, the more haste the less speed.

